# Precise frequency synchronization detection method based on the group quantization stepping law

**DOI:** 10.1371/journal.pone.0211478

**Published:** 2019-02-04

**Authors:** Baoqiang Du, Ran Deng, Xiyan Sun

**Affiliations:** 1 School of Electronic Information Engineering, Zhengzhou University of Light Industry, Zhengzhou, Henan, China; 2 School of Information and Communication, Guilin University of Electronic Technology, Guilin, Guangxi, China; Shandong University of Science and Technology, CHINA

## Abstract

A precise frequency synchronization detection method is proposed based on the group quantization steeping law. Based on the different-frequency group quantization phase processing, high-precision frequency synchronization can be achieved by measuring phase comparison result quantization. If any repeated phase differences in the quantized phase comparison results are used as the starting and stopping signal of the counter gate, the time interval between identical phase differences is a group period as gate time. By measuring and analyzing the quantized phase comparison results, the ±1−word counting error is overcome in the traditional frequency synchronization detection method, and the system response time is significantly shortened. The experimental results show that the proposed frequency synchronization detection method is advanced and scientific. The measurement resolution is notably stable and the frequency stability better than the E-12/s level can be obtained. The method is superior to the traditional frequency synchronization detection method in many aspects, such as system reliability and stability, detection speed, development cost, power consumption and volume.

## Introduction

The frequency difference between two signals is obtained by measuring the phase difference, and the high-precision frequency synchronization is realized. In recent years, regarding the traditional high-resolution phase-processing problem, to either optimize algorithm or improve the production process, the principle and processing method of phase measurement have not changed, but the measurement accuracy has been improved. K. Klepacki realized the phase difference measurement with 7.5 ps resolution by combining high-frequency pulse filling and fine delay [1], David Vyhlidal et al. improved this method, made the resolution of the phase difference measurement reach 0.17 ps order of magnitude, and obtained the time interval measurement precision with 2.1 ps [2]. The improved work of David Vyhlidal broadened the range of phase difference measurement, improved the linearity of measurement, and reduced the development cost of the measurement system. However, there is a ±1−word counting error in the phase difference measurement. B. Markovic converted the measured phase difference into a digital voltage, and measured the phase difference by measuring the digital voltage [3–4]. This method has the advantages of large dynamic range and easy integration, but the measurement resolution is limited by the conversion rate and digits of digital signals. This method can achieve 1.12 ps measurement resolution. L.Kostyantyna measured the phase difference between two comparison signals by using the phase coincidence detection method [5–6]. The method has high resolution, but the phase processing must be based on two signals of identical frequency. The phase comparison between signals with different frequencies requires complex frequency conversion processes such as mixing and frequency doubling, which normalizes the frequency, increases the development cost, introduces additional noise of the synthetic circuit and limits the universality of its application. This method can measure the phase difference better than the ps-level resolution and obtain the frequency stability with the E-13/s level. It is the most popular and effective ultra-high-resolution phase difference measurement method in the world. The measurement accuracy of this method is limited by the noise of the amplifier and mixer, particularly when the beat frequency is relatively low, the beat signal should be a rectangular wave to facilitate the time interval measurement. The effect of noise causes great difficulties in further improving the measurement accuracy.

With the development of aerospace, laser ranging, precision positioning, particle flight detection and other high-tech fields, there are higher requirements for the measurement accuracy of frequency signals, particularly radio frequency signals [7–9]. The commonly used frequency measurement methods include the direct counting method, multi-period synchronization method, analog interpolation method, and time Vernier method [10–11]. The direct counting method and the multi-period synchronous method have a ±1−word counting error [12–13]. Since the frequency of the filling signal is generally less than 10^9^Hz, the accuracy of the frequency measurement is usually lower than 10^−9^/s. The frequency measurement system designed by this method has the advantages of simple structure and low cost, but its accuracy is poor. The analog interpolation method has the ±1−word counting error, but it can reduce the error to 1/1000 words using an interpolator, and the measurement accuracy can reach the 10^−11^/s magnitude [14–15]. The instrument produced by the method has good precision, but its complex circuit design and expensive cost limit its wide application [16–17]. The phase coincidence detection technology effectively reduces the ±1−word counting error in the frequency measurement, and makes the measurement accuracy reach the 10^−10^/s magnitude. However, the non-uniqueness and randomness of the phase coincidence points make it difficult to further improve the accuracy. Therefore, a new frequency measurement method is presented based on different-frequency phase quantization processing. This method combines the frequency relationship between two comparison signals and the variation law of phase difference with FPGA on-chip technology, which solves the problems in the frequency measurement based on the phase coincidence detection, simplifies the circuit structure, reduces the cost, and improves the stability of the system.

## Methods

The frequency signal is the most accurate physical quantity in nature [18–21]. Various experiments show that the phase difference between signals with two different frequencies repeats with the interval of the least common multiple period. A high-precision measurement of the frequency signal can be achieved by using the physical law [22–23].

Let the periods of the frequency signals *f*_1_ and *f*_2_ be *T*_1_ and *T*_2_ respectively, then the relationship between signals with two different frequencies is as follows.

$$T_{1}=BT_{\max c}$$ (1)

$$T_{2}=AT_{\max c}$$ (2)

$$T_{\min c}=ABT_{\max c}=AT_{1}=BT_{2}$$ (3)

Where *A* and *B* are positive integers of mutual prime and *A*>*B*, *T*_max*c*_ is the greatest common factor period, and *T*_min*c*_ is the least common multiple period.

When there is no frequency deviation between two frequency signals, the phase difference conditions in *T*_min*c*_ are shown in formula (4).

$$\left[\begin{array}{l} P_{1}\\ P_{2}\\ \;\vdots \\ P_{B} \end{array}\right]=\left[\begin{array}{l} n_{1}T_{1}-T_{2}\\ n_{2}T_{2}-2T_{2}\\ \;\vdots \\ AT_{1}-BT_{2} \end{array}\right]$$ (4)

Formula (4) indicates that *P*_1_, *P*_2_,…, and P_B_ represent the phase difference conditions in *T*_min*c*_, and the last phase difference is *AT*_1_−*BT*_2_ = 0.

When there is a frequency deviation between two frequency signals, let $T_{2}^{\prime }=T_{2}+\Delta t$; the phase difference conditions in *T*_min*c*_ are as follows.

The phase difference variation in the first least common multiple period is shown in formula (5).

$$\left[\begin{array}{l} P_{1}^{\prime }\\ P_{2}^{\prime }\\ \;\vdots \\ P_{B}^{\prime } \end{array}\right]=\left[\begin{array}{l} n_{1}T_{1}‑1T_{2}^{\prime }\\ n_{2}T_{1}‑2T_{2}^{\prime }\\ \;\vdots \\ AT_{1}‑BT_{2}^{\prime } \end{array}\right]=\left[\begin{array}{l} n_{1}T_{1}‑T_{2}+\Delta t\\ n_{2}T_{1}‑2T_{2}+\Delta t\\ \;\vdots \\ \;B\Delta t \end{array}\right]$$ (5)

The phase difference variation in the second least common multiple period is shown in formula (6).

$$[\begin{array}{c} p_{1}^{\prime \prime }\\ P_{2}^{\prime \prime }\\ \vdots \\ P_{B}^{\prime \prime } \end{array}]=\left[\begin{array}{l} n_{1}T_{1}‑T_{2}+\Delta t+B\Delta t\\ n_{2}T_{1}‑2T_{2}+2\Delta t+B\Delta t\\ \;\vdots \\ \;2B\Delta t \end{array}\right]$$ (6)

The phase difference variation in the third least common multiple period is shown in formula (7).

$$\left[\begin{array}{l} p_{1}^{\prime \prime \prime }\\ P_{2}^{\prime \prime \prime }\\ \;\vdots \\ P_{B}^{\prime \prime \prime } \end{array}\right]=\left[\begin{array}{l} n_{1}T_{1}‑T_{2}+\Delta t+2B\Delta t\\ n_{2}T_{1}‑2T_{2}+2\Delta t+2B\Delta t\\ \;\vdots \\ \;3B\Delta t \end{array}\right]$$ (7)

The phase difference variation in the (n-1)th least common multiple period is shown in formula (8).

$$\left[\begin{array}{l} p_{1}^{\left(n-1\right)}\\ P_{2}^{\left(n-1\right)}\\ \;\vdots \\ P_{B}^{\left(n-1\right)} \end{array}\right]=\left[\begin{array}{l} n_{1}T_{1}‑T_{2}+\Delta t+(n‑2)B\Delta t\\ n_{2}T_{1}‑2T_{2}+2\Delta t+(n‑2)B\Delta t\\ \;\vdots \\ \;(n-1)B\Delta t \end{array}\right]$$ (8)

The phase difference variation in the nth least common multiple period is shown in formula (9).

$$\left[\begin{array}{l} p_{1}^{\left(n\right)}\\ P_{2}^{\left(n\right)}\\ \;\vdots \\ P_{B}^{\left(n\right)} \end{array}\right]=\left[\begin{array}{l} n_{1}T_{1}‑T_{2}+\Delta t+(n‑1)B\Delta t\\ n_{2}T_{1}‑2T_{2}+2\Delta t+(n‑1)B\Delta t\\ \;\vdots \\ \;nB\Delta t \end{array}\right]$$ (9)

From formula (5) to formula (9), we can obtain the scientific variation law of phase difference and group quantization in different-frequency phase-processing method. In any two contiguous least common multiple period, the stepping value of any phase difference is Δ*t*, $P_{B}^{\left(n-1\right)}-P_{B}^{\left(n\right)}=\Delta t,\;B\geq 2$, and the phase stepping value of group quantization is BΔt. When the stepping value of any phase difference *nB*Δ*t* = *T*_2_ in the entire phase comparison, any phase difference and group quantization will periodically change and repeatedly return to the initial state, turn round and round in turn. The experienced time that any phase difference and group quantization periodically change is called group period *T*_*gp*_, which is written as follows,

$$T_{gp}=n(T_{\min c}+B\Delta t)$$ (10)

Thus, by observing the repetition time of two phase differences, we can obtain the group period, and time variation Δ*t* can be calculated according to the stepping value of phase difference *nB*Δ*t* = *T*_2_ and formula (10).

$$\Delta t=\frac{T_{1}}{nB}=\frac{T_{1}T_{\min c}}{nT_{\min c}B}=\frac{T_{1}T_{\min c}}{T_{gp}B}$$ (11)

The true value of the reference signal period can be obtained using formula (11),

$$T_{2}^{\prime }=T_{2}+\Delta t=T_{2}+\frac{T_{2}T_{\min c}}{T_{gp}B}$$ (12)

T_2_ in formula (12) is the rough measurement result of the measured signal, *T*_min*c*_ is the least common multiple period between the coarse measurement value *T*_2_ and the frequency standard *T*_1_, and *T*_*gp*_ is the time that it takes for two identical phase differences to occur. After Δt is calculated, the actual measured signal $T_{2}^{\prime }$ can be obtained from $T_{2}^{\prime }=T_{2}+\Delta t$. Thus, the relationship between two signals can be expressed by the phase comparison results between two different-frequency signals.

**Fig 1:**
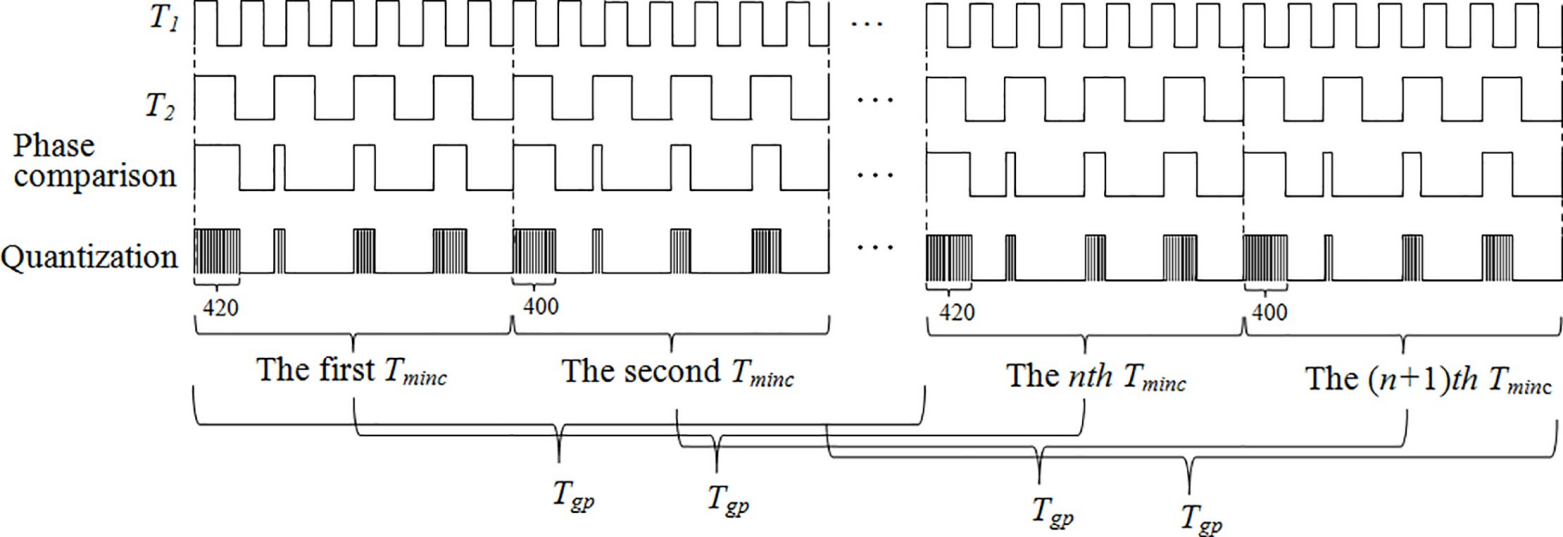
Group quantization phase processing.

Formula (12) indicates that it has a large measurement error. When the phase comparison results are counted, because of the counting error of ±1 words, the actual measurement result is an integral multiple of the group period. This error is not allowed to occur in the high-precision frequency measurement. By the analysis of the least common multiple period and group period, the regularity of the phase difference occurrence is obtained. The time between the appearances of two identical phase differences is a group period, as shown in Fig 1.

In a group period, *T*_1_ and $T_{2}^{\prime }$ are counted and the counting results are *m*_1_ and *m*_2_, respectively. Therefore, formula (13) can be obtained from the counting results of two comparison signals.

$$m_{1}T_{1}=m_{2}T_{2}^{\prime }$$ (13)

Fig 1 shows that the first phase difference in the first *T*_min*c*_ is equal to the first phase difference in the *n*th *T*_min*c*_ and the time that the phase difference recurs is only a group period.

If the counting value of the first phase difference in the first *T*_min*c*_ and the counting value of the first phase difference in the *n*th *T*_min*c*_ are used as the starting signal and stopping signal of the gate, respectively, and the two comparison signals are counted in the gate time, the frequency of the measured signal can be easily obtained using formula (13).

The measurement errors caused by phase quantization are analyzed as follows. According to the regularity of the phase comparison results between two different-frequency signals, the precise frequency linking between two comparison signals can be obtained, which makes the period of the measured signal be measured by the frequency standard signal.

Because of the repeated appearance of the same phase difference caused by the inherent law between two frequency signals, the real-time phase difference can be easily obtained through the different-frequency phase comparison and detection of phase comparison results using a high-frequency clock. The phase difference is used to produce the gate signal of the counter by simultaneously counting frequency standard signal, measured signal and phase difference. By counting the frequency standard signal and measured signal in the gate time, we can overcome the measurement errors caused by the counting error. If there is a ±1−word counting error in the counting results of the phase difference, the period of the stopping signal must be an integer multiple of the group period because of the 50% probability of the ±1−word counting error. Formula (13) is transformed into formula (14).

$$km_{1}T_{1}=km_{2}T_{2}^{\prime },k\in N$$ (14)

Formula (14) shows that even if there is a ±1−word counting error in the phase difference counting, the frequency measurement results of the measured signal are not affected. However, the gate time is extended because of the ±1−word counting error, which increases the measurement time and reduces the measurement speed.

## Discussion

**Fig 2:**
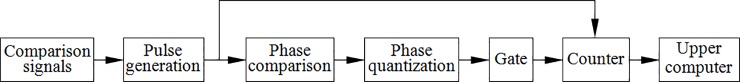
Frequency measurement scheme based on the group quantization phase processing.

According to the measurement principle based on phase quantization processing, the key to improving the measurement accuracy is the acquisition of phase comparison results and phase difference measurement in phase comparison results. The phase comparison results are obtained by detecting the phases of two processed signals. Then, the rising and falling edges of the phase comparison results are extracted, and the high-frequency clock is counted between the rising and falling edges of the phase comparison results. The two same counting results are used as the starting and stopping signals, and the frequency standard signal and measured signal are counted in the gate time. Finally, the counting results are sent to the upper computer for data processing and display. The system design scheme is shown in Fig 2.

The measured signal is a sine wave signal with 3.3V amplitude, which is generated by the signal generator KEYSIGHT E8663D and sent to FPGA by the global clock pin of the FPGA chip. The frequency standard signal is produced by the high-stability crystal oscillator OCXO BVA8607B-M and sent to FPGA by the global clock pin of the FPGA chip. The two signals with 100 MHz and 1 GHz are generated by the phase-locked loop in FPGA. The measured signal and frequency standard signal are transformed into narrow pulse signals by a delay of 100 MHz signal. Two narrow pulse signals are sent to phase detection modules with different frequencies to generate the phase comparison results with a group period as the cycle period, which consists of the least common multiple period. The phase comparison results are quantized by a high-frequency clock generated by the phase-locked loop in the FPGA and all phase differences in the phase comparison results are counted. The end time of any phase difference in the phase comparison results is used as the starting signal of the counter, and the time that the same phase difference reappears is used as the stopping signal of the counter. The frequency standard signal and measured signal are counted in the gate time, which basically completes a measurement. The counting results of two comparison signals are sent to the upper computer by a serial communication module generated by FPGA. The frequency measurement result of the measured signal is calculated by formula (13) on the upper computer.

### Pulse conversion

**Fig 3:**
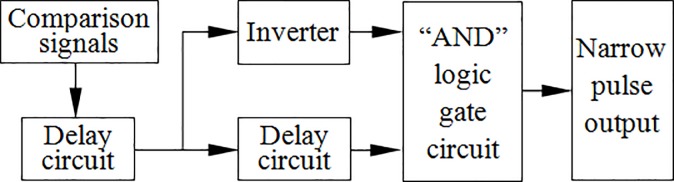
Pulse conversion scheme.

Because the FPGA can directly identify sine wave signals, the two comparison signals are introduced into the FPGA through the clock pins of the FPGA to transform the sine wave signal into a square wave pulse signal. The pulse conversion scheme is shown in Fig 3.

**Fig 4:**
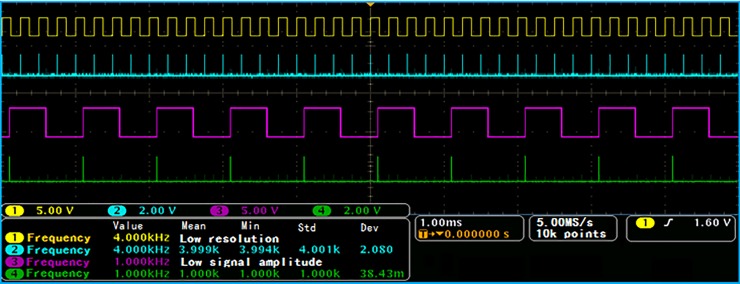
Pulse conversion test results.

In Fig 3, in addition to the delay function, the delay circuit also has the function of eliminating the noise of the comparison signals. The delay circuit is composed of the D trigger in the FPGA to make the pulse width equal to the delay length. The original square wave signal is delayed, and the delayed signal is sent to the inverter to obtain a signal that is opposite to the phase of the original square wave signal. The two processed square wave signals are sent to the “AND” logic gate circuit to obtain a narrow pulse signal with identical frequency to the original square wave signal. The specific test results are shown in Fig 4.

### Phase comparison circuit

**Fig 5:**
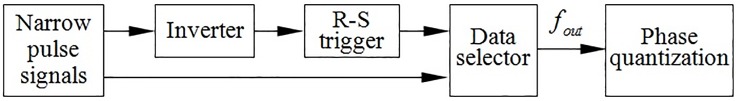
Direct phase comparison scheme.

The specific scheme of the direct phase comparison is shown in Fig 5.

**Fig 6:**
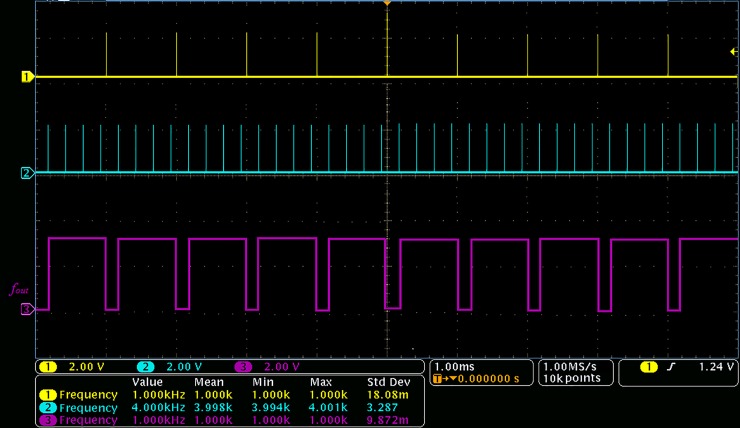
Direct phase comparison test results.

After the pulse transformation, two comparison signals are simultaneously converted into narrow pulse signals with identical frequency. This is not only conducive to the processing of phase comparison, but also greatly reduces the instability caused by the long rising time of sine wave. Thus, the phase detection becomes more stable. According to the scheme in Fig 5, the results of the direct phase comparison *f*_*out*_ are shown in Fig 6.

### Group quantization processing

**Fig 7:**
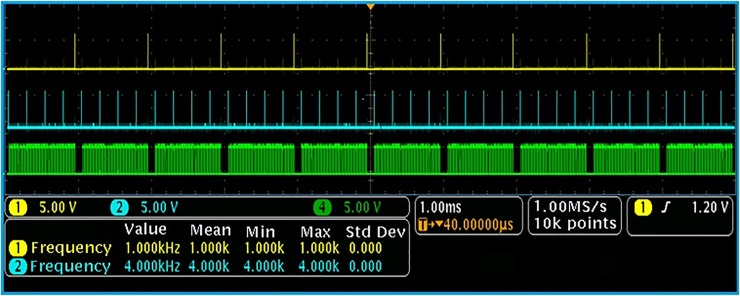
Group quantization test results.

To more conveniently quantize the phase comparison results and select the gate signal, a high-frequency clock is used in the FPGA, as shown in Fig 7.

Fig 7 shows that every phase difference in the phase comparison results is quantized. The quantization clock is generated by the phase-locked loop in the FPGA. The high-frequency clock is counted in the high level of phase comparison, and the time with two identical counting results is used as the gate signal of the counter.

## Results

According to the scheme in Fig 2, the frequency synchronization system prototype has been developed and tested. The frequency measurement range is 0.1–300 MHz. Frequency synchronization accuracy superior to the E-12/s level can be realized.

### Synchronization precision with the same source

The implementation of this system is based on the Cyclone-II-series FPGA chip produced by Altera Company. A frequency synthesizer E8663D is used to generate the measured signal. The external frequency standard of this frequency synthesizer comes from a high-stability crystal oscillator OCXO8607-BM 10MHz. The frequency synchronization detection results are shown in Table 1.

**Table 1:** Frequency synchronization detection results with the same source.

Measured frequency	Frequency difference	Synchronization precision
300 MHz	0.230783 Hz	4.74×10^−15^/s
250 MHz	0.006812 Hz	4.19×10^−15^/s
200 MHz	0.060449 Hz	3.67×10^−15^/s
150 MHz	0.700371 Hz	3.31×10^−15^/s
100 MHz	0.350592 Hz	3.15×10^−15^/s
50 MHz	0.005834 Hz	2.78×10^−15^/s
10 MHz	0.030623 Hz	2.43×10^−15^/s
5 MHz	0.000358 Hz	2.24×10^−15^/s

### Synchronization precision with different sources

To verify the frequency synchronization precision of the system prototype, the ultra-high-stability crystal oscillator OCXO H800-U 10 MHz signal is used as the system external frequency standard in the frequency measurement experiment. A KEYSIGHT E8663D frequency synthesizer that uses the OCXO BVA8607BM 10 MHz frequency signal as the external frequency standard is used to produce the measured signal. The specific measurement results are shown in Table 2.

**Table 2:** Frequency synchronization detection results with different sources.

Measured frequency	Measuring difference	Synchronization precision
300 MHz	0.040223 Hz	6.26×10^−12^/s
250 MHz	0.010652 Hz	5.68×10^−12^/s
200 MHz	0.100335 Hz	4.91×10^−12^/s
150 MHz	0.303126 Hz	4.64×10^−12^/s
100 MHz	0.005897 Hz	4.13×10^−12^/s
50 MHz	0.004571 Hz	3.62×10^−12^/s
10 MHz	0.006759 Hz	5.11×10^−13^/s
5 MHz	0.008574 Hz	4.87×10^−13^/s

From Table 1, the frequency synchronization precision of the system can reach the E-15/s level. Because the generation of the frequency standard signal and the synthesis of the measured signal are from the same ultra-high stability crystal oscillator, they have identical frequency drifts. Therefore, the effect of the frequency source phase noise on the system error is suppressed. A higher frequency stability can be obtained because of noise cancellation in the same frequency source. In traditional frequency synchronization detection methods, the double mixing time difference method is the most widely used and accurate frequency measurement method in the world. The frequency measurement range of the dual mixing time difference method is generally 1 MHz– 30 MHz. To widen the frequency measurement range, a certain number of mixers must be added. Additional noise superposition introduced by the mixer and frequency normalization circuit increases the jitter of the gate switching signal and decreases the system frequency stability. Therefore, it is very difficult to obtain the wide frequency range and high frequency stability simultaneously in this method. For any frequency signal in the radio frequency range, the frequency stability of the system can reach the E-14/s level, for example, for 10 MHz and 5 MHz frequency signals, the frequency stability of the system can reach the 2.5E-14/s and 5E-14/s levels, respectively, which reduces an order of magnitude compared with the data in Table 1. Because the double mixing time difference method mainly relies on the frequency difference multiplication effect to improve the frequency stability, as long as the measurement resolution of the time interval counter meets 25 ns, 40 MHz clock frequency, the frequency stability of the system can reach the 2.5E-14/s level. However, it is difficult to further improve the system measurement accuracy because of the limitation of the counter clock frequency and the ±1− word counting error. In engineering practice, the double mixing time difference system has a complex structure, huge volume, high development cost and high market price, which seriously limits its wide applications.

From Table 2, the frequency synchronization precision of the system can reach the E-13/s level. The two comparison signals are from different frequency sources. The phase noise of the two frequency sources has a superposition effect on the measurement error of the system, which makes the phase difference jitter after the phase comparison. The counting of the two comparison signals produces errors in the gate time. The counting results with errors are sent to the host computer, and the system frequency stability decreases because of the data processing in the host computer. However, because the phase coincidence detection is not used in the frequency measurement, the effect of the system measurement error is restrained to a certain extent.

In engineering applications, the effect of additional noise on the frequency synchronization precision is reduced by improving the clock frequency of the FPGA system and optimizing the upper computer algorithm. Then, the detection results superior to the E-13/s level can be reached.

## Conclusions

The proposed high-precision frequency synchronization detection method based on the group quantization stepping law is no longer to use the traditional phase comparison method to improve the measurement precision by simply relying on the improvement of the line circuit or the development of microelectronic devices. The proposed method applies the inherent relations and changing laws of the periodic signals in nature to the mutual relationship processing among the frequency signals to complete the mutual phase comparison and processing without frequency normalization. According to the phase comparison law between signals with different frequencies, the gate is selected by the phase measurement, which overcomes the difficulty of finding phase coincidence points and the uncertainty and randomness of the gate controlled by the phase coincidence points. Experimental results show that the frequency stability can reach the E-13/s level. Compared with traditional frequency measurement methods such as the analog interpolation method, time Vernier method and phase comparison method [24], this method has the advantages of high measurement accuracy, simple circuit structure, low cost and high system stability. With the development of microelectronics technology and improved FPGA performance, the measurement accuracy of this frequency synchronization detection system may be further improved.

## Supporting information

S1 Table Frequency synchronization detection results with the same source. (DOCX)

S2 Table Frequency synchronization detection results with different sources. (DOCX)
