# Supplementary material for: Precise frequency synchronization detection method based on the group quantization stepping law
Source: PLoS One. 2019 Feb 4;14(2):e0211478. doi: 10.1371/journal.pone.0211478 (PMC6361435; doi:10.1371/journal.pone.0211478)
Supplement: S1 Table — (DOCX) [file pone.0211478.s001.docx]

Table 1. Frequency synchronization detection results with the same source.

| Measured frequency | Frequency difference | Synchronization precision |
| --- | --- | --- |
| 300 MHz | 0.230783 Hz | 4.74×10^-15^/s |
| 250 MHz | 0.006812 Hz | 4.19×10^-15^/s |
| 200 MHz | 0.060449 Hz | 3.67×10^-15^/s |
| 150 MHz | 0.700371 Hz | 3.31×10^-15^/s |
| 100 MHz | 0.350592 Hz | 3.15×10^-15^/s |
| 50 MHz | 0.005834 Hz | 2.78×10^-15^/s |
| 10 MHz | 0.030623 Hz | 2.43×10^-15^/s |
| 5 MHz | 0.000358 Hz | 2.24×10^-15^/s |
