# Supplementary material for: Precise frequency synchronization detection method based on the group quantization stepping law
Source: PLoS One. 2019 Feb 4;14(2):e0211478. doi: 10.1371/journal.pone.0211478 (PMC6361435; doi:10.1371/journal.pone.0211478)
Supplement: S2 Table — (DOCX) [file pone.0211478.s002.docx]

Table 2. Frequency synchronization detection results with different sources.

| Measured frequency | Measuring difference | Synchronization precision |
| --- | --- | --- |
| 300 MHz | 0.040223 Hz | 6.26×10^-12^/s |
| 250 MHz | 0.010652 Hz | 5.68×10^-12^/s |
| 200 MHz | 0.100335 Hz | 4.91×10^-12^/s |
| 150 MHz | 0.303126 Hz | 4.64×10^-12^/s |
| 100 MHz | 0.005897 Hz | 4.13×10^-12^/s |
| 50 MHz | 0.004571 Hz | 3.62×10^-12^/s |
| 10 MHz | 0.006759 Hz | 5.11×10^-13^/s |
| 5 MHz | 0.008574 Hz | 4.87×10^-13^/s |
